# Proteome Profiles of Digested Products of Commercial Meat Sources

**DOI:** 10.3389/fnut.2017.00008

**Published:** 2017-03-27

**Authors:** Li Li, Yuan Liu, Guanghong Zhou, Xinglian Xu, Chunbao Li

**Affiliations:** ^1^Key Laboratory of Meat Processing and Quality Control, MOE, Jiangsu Synergetic Innovation Center of Meat Processing and Quality Control, Nanjing Agricultural University, Nanjing, China; ^2^Key Laboratory of Animal Products Processing, MOA, Jiangsu Synergetic Innovation Center of Meat Processing and Quality Control, Nanjing Agricultural University, Nanjing, China; ^3^College of Food Science and Technology, Shanghai Ocean University, Shanghai, China

**Keywords:** meat products, *in vitro* digestion, particle size, digestibility, LC–MS/MS, oxidation

## Abstract

This study was designed to characterize *in vitro*-digested products of proteins from four commercial meat products, including dry-cured ham, cooked ham, emulsion-type sausage, and dry-cured sausage. The samples were homogenized and incubated with pepsin and trypsin. The digestibility and particle sizes of digested products were measured. Nano-LC–MS/MS was applied to characterize peptides. The results showed the highest digestibility and the lowest particle size in dry-cured ham (*P* < 0.05), while the opposite was for cooked ham (*P* < 0.05). Nano-LC–MS/MS analysis revealed that dry-cured ham samples had the greatest number of 750–3,500 Da Mw peptides in pepsin-digested products. In the digested products of cooked ham and emulsion-type sausage, a lot of peptides were matched with soy protein that was added in the formulations. In addition, protein oxidation was also observed in different meat products. Our findings give an insight into nutritional values of different meat products.

## Introduction

Meat processing has been shown to affect its quality, especially of protein digestibility and nutritional values (1). Different processes could induce meat proteins to different changes. For example, dry-cured ham is characteristic of long-term salting, drying, and aging (2, 3). Cooked ham and emulsion-type sausage are characteristic of tumbling, chopping, and cooking (4, 5). Dry-cured sausage is characteristic of mixing of muscle and fat particles and fermentation for a certain time (6). These procedures may cause significant differences in protein bioavailability in gastrointestinal tract. In most cases, meat products need to be cooked before eating. Cooking temperature and time had a significant influence on protein oxidation and aggregation (7, 8), and moderate degree of cooking is essential for efficient digestion in the gastrointestinal tract (9). However, protein aggregation systems of cooked ham and emulsion sausage are quite different in that the former is characterized mainly by disulfide bridges, and the latter is characteristic of covalent inter-protein links (10). In a recent study, we compared the *in vitro* digestibility of cooked pork, emulsion-type sausage, dry-cured pork, and stewed pork that were prepared by the same meat source and found significant differences in digestibility, digested products, and other attributes (11). In practice, commercial meat products may have greater variations in nutrition because of different meat source, formulations, and techniques. However, few data are available on protein digestibility of commercial meat products.

In recent years, *in vitro* digestion model and high-throughput LC–MS/MS technologies have been widely applied to simulate meat digestion in the stomach and the small intestine (9, 12). Proteolysis and protein oxidation were observed during processing of fermented meat products as a result of combined action of endogenous and microbial exopeptidases (13, 14). Protein oxidation induced the changes in hydrophobicity, protein aggregation, and secondary structure, which further had an influence on *in vitro* digestibility (15). Medium cooking may increase *in vitro* digestibility of meat proteins but prolonged cooking at 100°C would have a negative influence (16).

The present study was designed to compare *in vitro* digestibility and digested products of proteins from four commercial meat products by nano-LC-LTQ-Orbitrap XL MS/MS system.

## Materials and Methods

### Materials

#### Reagents

Porcine gastric pepsin (cat. no. P7125) and porcine pancreatic trypsin (cat. no. T7409) were obtained from Sigma-Aldrich (St. Louis, MO, USA). BCA protein assay kit (cat. no. 23225) and protein calibration marker ranging from 10 to 250 kDa (cat. no. 26619) were obtained from Thermo Scientific (Rockford, IL, USA). Amicon Ultracel-3 membrane (UFC500396) and ZipTip C18 pipette tips (ZTC18S096) were obtained from Millipore (Billerica, MA, USA).

#### Samples

Four meat products (dry-cured ham, cooked ham, emulsion-type sausage, and dry-cured sausage) were obtained from a commercial meat company (Yurun Group, Nanjing). According to the procedures from the company, dry-cured hams were processed by salting, soaking and washing, sun-drying, loft-aging, and post-aging over a period of 9 months (3). Smoked cooked ham was prepared mainly with pork hind-leg muscles and soy protein (total protein content: 19.7%) and processed by curing, tumbling, stuffing, cooking, and slicing (17). Emulsion-type sausages were made with hind-leg muscles and soy protein by chopping, stuffing, and cooking. Dry-cured sausage was processed with hind-leg muscles by mincing, mixing, stuffing, drying, and fermentation. The dry-cured sausage was then cooked and packed. For each product, seven repetitions were used.

### *In Vitro* Digestion

Meat products were *in vitro* digested according to Wen et al. (9) with a slight modification. Briefly, 100 g of meat samples were chopped, and 0.5 g of sample was homogenized with a homogenizer (Ultra Turrax T25 Basic, IKAWerke, Staufen, Germany) in 2 mL of phosphate buffer solution (10 mmol/L ${\text{Na}}_{2}{\text{HPO}}_{4}^{-}$ NaH_2_PO_4_, pH7.0) for 2 s× 30 s at 9,500 rpm and 2 s× 30 s at 13,500 rpm with 30 s cooling between bursts at 4°C.

The homogenates were adjusted to pH 2.0 with 1 mol/L HCl, and pepsin digestion was initiated by adding gastric pepsin at a ratio of 1–31.25 on a meat mass basis (substrate). The digestion was maintained at 37°C for 2 h and stopped by adding 1 mol/L NaOH to adjust the pH to 7.5. Afterward, trypsin was added to the resulting mixture of gastric digestion at a ratio of 1–50 on the substrate basis. The digestion was kept at 37°C for 2 h and stopped by heating the mixture at 95°C for 5 min. After pepsin and trypsin digestion, the undigested proteins were precipitated by adding threefolds of ethanol (V:V) and staying at 4°C for 12 h and removed by centrifugation at 10,000 × *g* for 20 min at 4°C. The supernatant was stored at −18°C for peptide identification.

The *in vitro* digestibility was calculated as the difference in protein contents before and after digestion. Two aliquots of meat samples were taken from each sample. One aliquot was digested with pepsin, and the other one was digested with pepsin and subsequently with pancreatic trypsin. The digestion procedures were the same as above. After digestion, the resulting mixtures were centrifuged at 10,000 × *g* for 20 min at 4°C and the supernatant was discarded. The protein contents in the precipitates and the untreated products were determined by the BCA protein assay kit (Thermo Scientific, Rockford, IL, USA) according to the manufacturer’s instructions. The degree of digestibility was calculated as follows:

$$\text{Digestibility}\left(\%\right)=\left(W_{0}-W_{1}\right)/W_{0}\times 100$$

where *W*_1_ is protein content (g) in the precipitate after pepsin or trypsin digestion and *W*_0_ is protein content (g) in the untreated product before digestion.

### Peptide Identification of Digested Products

The ethanol-soluble fractions of enzyme-digested products were identified, as described by Wen et al. (9) with minor modifications. Briefly, the ethanol-soluble fractions were transferred to ultra-0.5 mL centrifugal filter units (Amicon Ultra, Millipore, Billerica, MA, USA) and centrifuged at 15,000 × *g* for 15 min. The filtrates were cleaned/concentrated with ZipTip C18 (Millipore, Billerica, MA, USA). After that, the concentrated peptide mixture was loaded onto a C18 column (2 cm × 200 μm, 5 μm) and then passed through a C18 chromatographic column (75 μm × 100 mm, 3 μm) for separation. Peptides were separated by running a mobile phase changing from 0.1% formic acid in water (buffer A) to 0.1% formic acid in 84% acetonitrile (buffer B). A step-gradient elution at a flow rate of 300 nL/min was applied with a gradient elution: 0–12 min (97%A, 3%B), 12–100 min (72%A, 28%B), 100–120 min (45%A, 55%B), 122–144 min (2%A, 98%B), and 144–160 min (97%A, 3%B). The hybrid quadrupole orbitrap mass spectrometer equipped with a nanoelectrospray ionization source (Thermo Fisher Scientific, USA) was operated in a data-dependent mode, and a scan cycle was initiated with a full-scan MS spectrum (from 750 to 3,500 amu).

MS/MS spectra of peptides were matched using the Proteome Discoverer-1.4 (Thermo Fisher Scientific, Palo Alto, CA, USA) against the Swiss-Prot database against *Sus scrofa* for pork and Glycine max for soy protein.^1^ Data matching was performed with a parent ion tolerance of 10 ppm, oxidation of methionine was chosen as dynamic modifications, and two missing cleavages were allowed. Pepsin was used in peptic peptides database search, while both pepsin and trypsin were used in tryptic peptides search. Venn diagrams were applied to analyze the similarity of peptides from the four meat products.^2^

### Measurement of Particle Size before and after Digestion

The homogenization and *in vitro* digestion of meat products were performed, as described above. Particle sizes of homogenates before and after digestion were measured by an integrated laser light scattering instrument (Mastersizer 3000, Malvern, Worcestershire, UK). The parameters were set as follows: type of particle, opaque; density (grams per square centimeter), 0.95; dispersing agent, water; index of refraction, 1.414; and sensor threshold, 100. The data were analyzed by using the Malvern Mastersizer software (version 5.12c, Malvern, Worcestershire, UK). Several parameters were achieved, including *D*_4,3_ representing the mean diameter in volume, *D*_3,2_ for the mean diameter in surface, *D_x_*_(50)_ for the size for which 50% of the sample particles have a lower size, *D_x_*_(10)_ for the size for which 10% of the sample particles have a lower size, and *D_x_*_(90)_ for the particle size for which 90% of the sample particles have a lower size.

### Statistical Analysis

One-way analysis of variance and Duncan’s multiple-range test were performed to test the difference in digestibility and particle size among four meat products with the SAS program (version 9.1.2, 2008, SAS Institute Inc., USA).

## Results

### The *In Vitro* Digestibility

**Figure 1:**
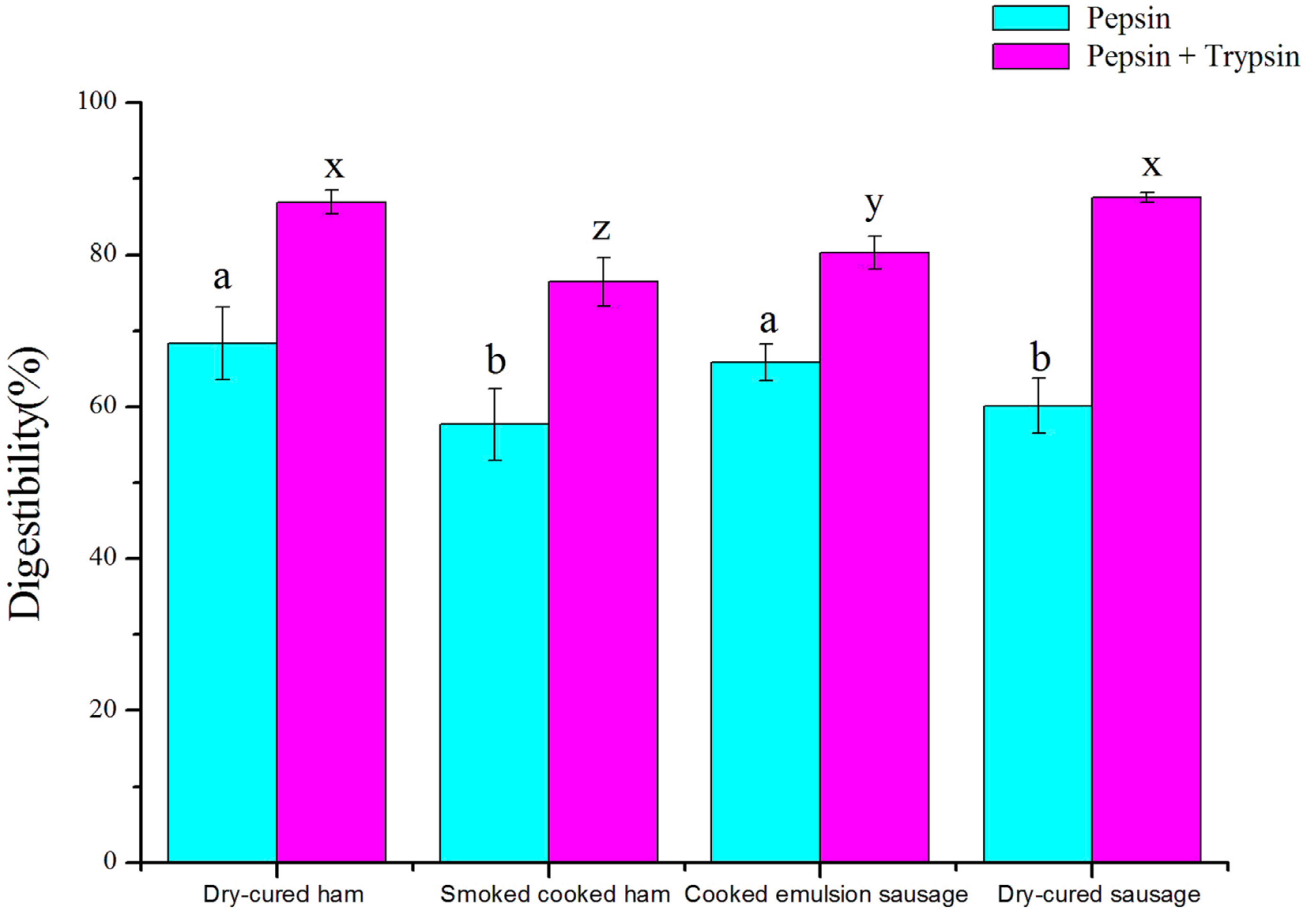
***In vitro* protein digestibility of four meat products (means ± SDs, *n* = 7)**. a, b, different letters indicated significant differences in digestibility after pepsin digestion; x, y, z, different letters indicated significant differences in digestibility after trypsin digestion.

There were significant differences in protein digestibility among four commercial meat products (*P* < 0.05, Figure 1). Cooked ham had the lowest *in vitro* digestibility under both pepsin and trypsin digestion conditions (*P* < 0.05), while cooked dry-cured ham showed the highest digestibility (*P* < 0.05). Dry-cured sausage showed lower digestibility than emulsion sausage (*P* < 0.05) under pepsin digestion, but the results were reverse after trypsin digestion (*P* < 0.05).

### Particle Size

Dry-cured ham showed the lowest values of all parameters, while the highest values for smoked cooked ham (Table 1, *P* < 0.05). Particle sizes of homogenates decreased greatly after pepsin digestion (Table 1, pepsin digested vs. undigested). Dry-cured ham still had the lowest values of all parameters, and again, the highest values were observed for smoked cooked ham. After trypsin digestion, particle sizes of cooked ham and dry-cured sausage further decreased compared to their pepsin-digested counterparts (Table 1, pepsin + trypsin digested vs. pepsin digested). Again, dry-cured ham kept the smallest particle size but the largest for cooked ham (*P* < 0.05).

**Table 1:** **The particle size of four meat products (means ± SDs, *n* = 7)**.

	*D*_3,2_	*D*_4,3_	*D_x_*_(10)_	*D_x_*_(50)_	*D_x_*_(90)_
**Undigested samples**					
Dry-cured ham	5.27 ± 0.41^d^	67.66 ± 5.72^d^	2.06 ± 0.08^d^	10.67 ± 1.93^d^	182.70 ± 6.70^c^
Smoked cooked ham	37.03 ± 6.33^a^	287.88 ± 14.41^a^	19.88 ± 4.89^a^	278.38 ± 13.75^a^	603.75 ± 14.47^a^
Cooked emulsion sausage	16.83 ± 2.32^c^	105.41 ± 3.83^c^	6.24 ± 1.02^c^	55.50 ± 4.72^c^	278.13 ± 9.20^b^
Dry-cured sausage	21.03 ± 1.95^b^	254.50 ± 10.24^b^	10.12 ± 0.96^b^	202.25 ± 11.21^b^	595.13 ± 6.66^a^
**Pepsin-digested samples**					
Dry-cured ham	4.04 ± 0.22^c^	59.11 ± 3.51^c^	1.70 ± 0.19^c^	9.30 ± 1.09^d^	98.98 ± 7.10^c^
Smoked cooked ham	23.99 ± 4.02^a^	153.10 ± 11.25^a^	12.98 ± 1.19^a^	124.31 ± 7.36^a^	360.25 ± 11.59^b^
Cooked emulsion sausage	11.9 ± 1.19^b^	47.63 ± 4.53^d^	5.40 ± 0.5^b^	29.16 ± 4.21^c^	109.50 ± 9.86^c^
Dry-cured sausage	13.09 ± 2.29^b^	141.18 ± 12.28^b^	4.97 ± 1.11^b^	71.25 ± 7.52^b^	393.38 ± 13.77^a^
**Pepsin/trypsin digested samples**					
Dry-cured ham	5.19 ± 0.90^c^	23.18 ± 4.77^c^	1.83 ± 0.23^b^	10.92 ± 1.50^c^	49.16 ± 8.83^d^
Smoked cooked ham	14.16 ± 2.93^a^	66.43 ± 5.46^a^	5.57 ± 1.26^a^	45.69 ± 10.79^a^	191.25 ± 6.96^a^
Cooked emulsion sausage	13.66 ± 2.10^a^	35.08 ± 4.09^b^	6.47 ± 1.30^a^	28.35 ± 3.94^b^	70.35 ± 7.55^c^
Dry-cured sausage	7.43 ± 1.01^b^	64.75 ± 4.17^a^	2.57 ± 0.53^b^	27.20 ± 5.15^b^	174.39 ± 9.38^b^

*^a,b,c,d^Different superscripts indicate significant difference among different meat products*.

### Nano-LC-LTQ-Orbitrap XL MS/MS

**Figure 2:**
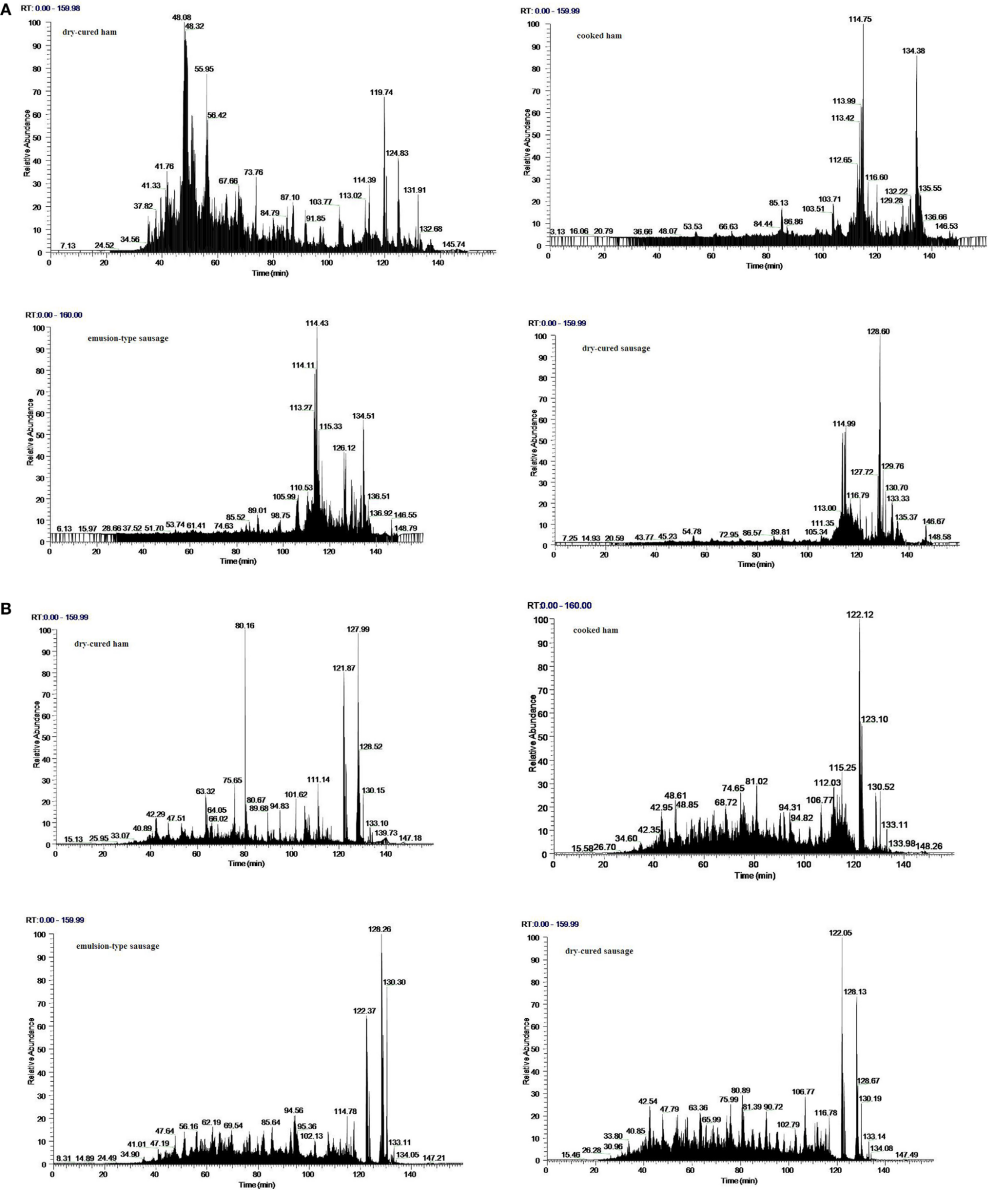
**Representative total ion chromatogram spectra of ethanol-soluble fragments from *in vitro* digested products of four meat products**. **(A)** *In vitro* digestion with pepsin; **(B)** *in vitro* digestion with pepsin followed by trypsin.

The ethanol-soluble fragments of four meat products were identified on a nano-LC*–*MS/MS system, and representative total ion chromatogram spectra were shown in Figure 2. These spectra only covered the peptides of molecular weights ranging from 750 to 3,500 Da within 160 min elution time. Generally, dry-cured ham showed large difference in peptide composition from the other three meat products after pepsin digestion (Figure 2A) and trypsin digestion (Figure 2B).

**Figure 3:**
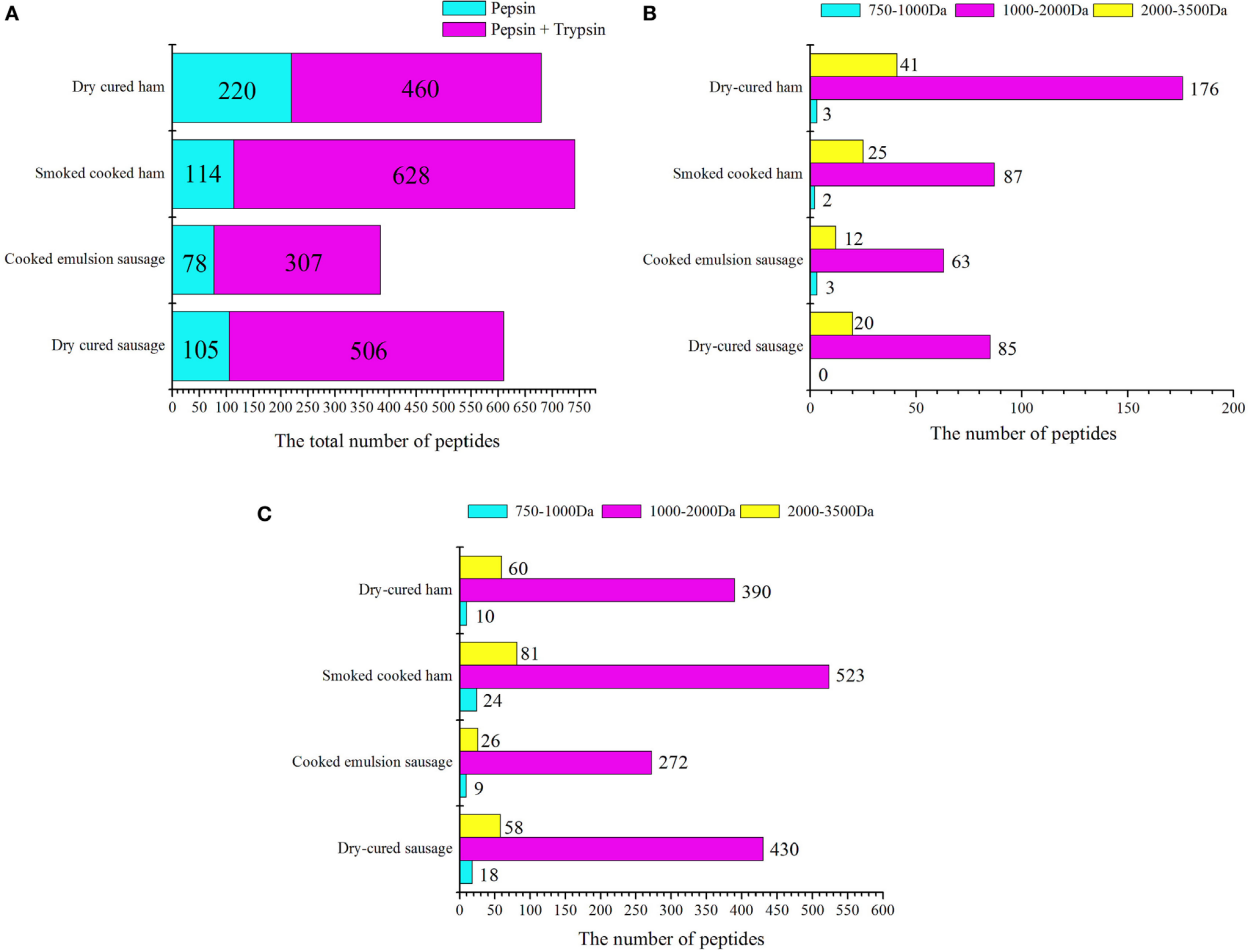
**Statistics of peptides from *in vitro*-digested products of four meat products**. **(A)** General statistics for pepsin and trypsin digesta. For cooked ham, 33 of 114 peptides in pepsin digesta were matched with soy proteins, and 80 of 628 peptides in pepsin/trypsin digesta were matched with soy proteins. For cooked emulsion sausage, 43 of 78 peptides in pepsin digesta and 132 of 311 peptides in pepsin/trypsin digesta were matched with soy proteins. **(B,C)** Statistics of peptides on the basis of molecular weights after pepsin and trypsin digestion, respectively.

In the pepsin digesta, dry-cured ham had the greatest number of peptides, 176 of which had molecular weights ranging from 1,000 to 2,000 Da, and 41 had molecular weights greater than 2,000 Da (Figure 3A; File S1 in Supplementary Material). Interestingly, many peptides were eluted from dry-cured ham samples at earlier time in the reverse phase LC system, indicating that these peptides are more hydrophobic (Figure 3B) and might be more susceptible to trypsin and other peptidases. Cooked ham and emulsion-type sausage had smaller number of peptides. In emulsion-type sausage samples, 35 of 78 peptides were matched with meat proteins, including myosin, metabolic enzymes, and other sarcoplasmic proteins, while the other 43 peptides were matched with soy proteins, including glycinin and conglycinin (File S1 in Supplementary Material). This could be accounted for high percentage of soy protein in the sausage formulation. In cooked ham, 81 of 114 peptides were matched with myofibrillar and sarcoplasmic proteins, while the other 33 peptides were matched with soy protein. In cooked dry-cured sausage, 105 peptides were matched with myosin and metabolic enzymes. These results indicate that meat proteins in cooked ham and sausage may not be susceptible to pepsin digestion due to gelling and emulsion structures. This is in accordance with the results of digestibility and particle sizes.

In the pepsin/trypsin digesta, a great number of peptides were matched with myofibrillar proteins, in particular to myosin. In addition, peptides from pepsin were also detected. The numbers of detectable peptides were increased by 2.1-, 5.5-, 3.9-, and 4.8-folds for dry-cured ham, cooked ham, emulsion-type sausage, and dry-cured sausage, respectively (Figure 3C). For cooked ham, 80 of 628 peptides were matched with soy proteins, while 132 of 307 peptides in emulsion-type sausage were matched with soy proteins (File S2 in Supplementary Material). Although the molecular weight range did not change too much, the increased number of peptides represented enhanced susceptibility of myofibrillar and sarcoplasmic proteins to trypsin digestion. On the other hand, pepsin-digested peptides could be further degraded by trypsin into smaller peptides that could not be detectable.

**Figure 4:**
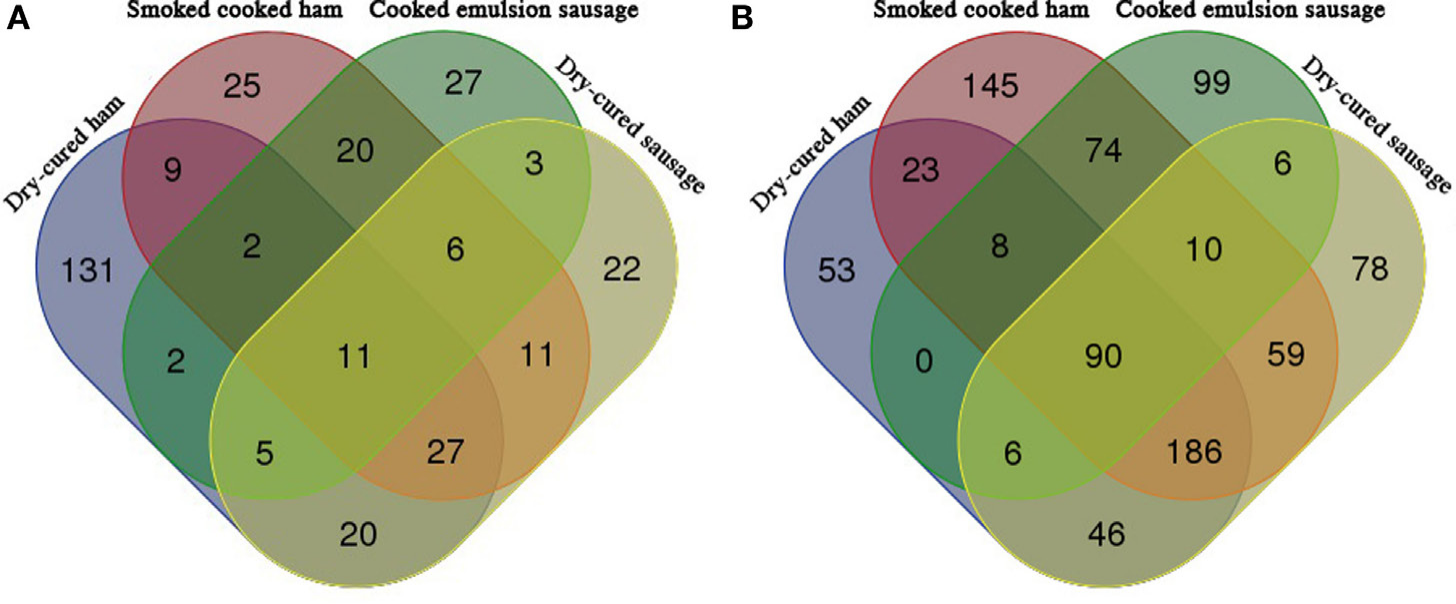
**Venn diagrams of peptides obtained from meat products (*n* = 7)**. **(A)** *In vitro* pepsin digesta. Ten of 25 cooked ham-specific peptides were matched with soy proteins, while 21 of 27 cooked emulsion sausage-specific peptides were matched with soy proteins. **(B)** *In vitro* pepsin/pepsin digesta. Fifteen of 145 cooked ham-specific peptides were matched with soy proteins, while 69 of 99 cooked emulsion sausage-specific peptides were matched with soy proteins.

Venn diagrams indicated that only 11 identified peptides were common for all the 4 commercial meat products in pepsin digesta, but 90 common peptides were identified common in pepsin/trypsin digesta (Figure 4). There were 131, 25, 27, and 22 peptides individually specific for dry-cured ham, cooked ham, emulsion-type sausage, and cooked dry-cured sausage in pepsin digesta (Figure 4A), and 53, 145, 99, and 78 peptides specific for pepsin/trypsin digesta, respectively (Figure 4B).

Nano-LC-LTQ-Orbitrap XL MS/MS also showed different levels of oxidation of the methionine residues in four commercial meat products. In pepsin digesta of dry-cured ham, 19 of 220 peptides exhibited methionine oxidation (File S1 in Supplementary Material), while the digesta of cooked ham and dry-cured sausage had only 1 peptide exhibiting methionine oxidation. The emulsion-type sausage did not show methionine oxidation. In trypsin digesta, 57 of 460 peptides showed methionine oxidation in dry-cured ham, with 32/628 for cooked ham, 9/307 for emulsion-type sausage, and 23/506 for cooked dry-cured sausage (File S2 in Supplementary Material).

## Discussion

In animal studies, dietary meat proteins have been shown to affect gut microbiota, metabolism in liver, and host health (18, 19). The quality of dietary meat proteins is quite important. If the proteins are oxidized seriously during processing, proteins will be difficult to be digested and absorbed in the small intestine and some of them will move to the cecum and colon. This will change the composition of gut microbiota and further gut health.

In a laboratory study, we observed significant differences in protein digestibility among stewed pork, dry-cured pork, emulsion-type sausage, and cooked pork and identified differential peptides by LC–MS/MS (11). On the factory level, the techniques may be quite different, which would result in different nutritional quality of meat products. For example, soy protein and starch could be added in the emulsion-type sausage formulations in practice in order to lower the cost and realize different texture attributes. However, in the laboratory study, we did not add such ingredients. The processing of dry-cured ham is highly costly and time consuming in practice, and thus in our previous study, we just used dry-cured pork to mimic. And therefore, we focused the protein digestibility of four commercial meat products.

The differences in *in vitro* digestibility among commercial meat products could be interpreted for two aspects: (1) proteolysis under the endogenous enzymes (e.g., lysosomes, animopeptidases, carboxypeptidases, dipeptidases, and tripeptides) during processing could contribute to the relatively high digestibility of dry-cured ham (2, 3, 6, 14). (2) For cooked ham, salt-soluble proteins (i.e., myofibrillar proteins) could be extracted during long-term tumbling, and gelling may occur during subsequent cooking. It is known that intermolecular cross-links are formed between myosin molecules and other proteins, which form close networks (20). These networks are necessary to produce good texture, but they may be partially resistant to enzymatic digestion. For emulsion-type sausage, the fat droplets around muscle fibers in the emulsion system (21) might affect enzymatic digestion of myofibrillar proteins. During cooking, protein oxidation and aggregation may directly or indirectly affect proteolytic susceptibility of myofibrillar proteins to pepsin (7). In addition, cooking temperature is critical for the proteolytic susceptibility of myofibrillar proteins to pepsin and/or trypsin. Moderate denaturation of meat proteins at appropriate temperature (70°C) could accelerate pepsin digestion due to the exposure protein cleavage sites accessible to enzymes; however, higher temperature (above 100°C) would increase protein oxidation and aggregation but decelerate pepsin digestion (8).

Our results indicated that dry-cured ham had the smallest particle sizes, but the largest for smoked cooked ham. The differences in particle sizes could be attributed to biochemical and physical changes. In dry-cured ham, proteolysis during long-term aging would promote the transformation of high molecular weight proteins into polypeptides and amino acids, accompanying with the decline of particle sizes (22). In addition, salting and drying may induce the increase in protein surface hydrophobicity and the “condensing effect” of molecular structure of the proteins due to oxidation and aggregation (23). In dry-cured sausage, relatively strong proteolysis could take place under both endogenous and microbial enzymes (14). However, protein oxidation and aggregation also extensively happen to dry-cured sausage, and a significantly negative correlation has been observed between gastric pepsin activity and carbonyl group formation, S–S groups, protein surface hydrophobicity, and *D*_4,3_ (15). As described above, in cooked ham and cooked emulsion sausage, gelling network structure and emulsion system may, to a certain context, affect pepsin digestion and particle size. In addition, the number and the size of particles would reduce as cooking time and temperature increase (23).

Long duration of processing may cause relatively high level of protein oxidation, and the oxidized protein seemed resistant to pepsin digestion but not to trypsin digestion. It has been reported that methionine in 6–35% peptides derived from myofibrillar proteins of dry-cured ham, including nebulin, titin, myosin heavy chains, LIM domain-binding protein 3, and troponin I proteins, were oxidized during dry-curing process (2). However, this kind of oxidation seems not associated with particle size and digestibility. It is the formation of disulfide bridges due to the oxidation of cysteine, dityrosine bridges due to the oxidation of tyrosine and other bonds during processing and cooking that affect protein digestion because these bonds are the key force to protein aggregation (23).

In summary, four commercial meat products had *in vitro* protein digestibility. Dry-cured ham had the highest protein digestibility and the smallest particle size, while it was the opposite for cooked ham. Nano-LC–MS/MS revealed that proteins in cooked ham and emulsion-type sausage showed lower susceptibility to pepsin digestion in terms of the numbers of detectable peptides ranging from 750 to 3,500 Da. However, higher level of methionine oxidation occurred in dry-cured ham. Gelling network and emulsion system might cause the lower digestibility for cooked ham and emulsion-type sausage.

## Author Contributions

CL and GZ designed the research; LL, YL, and XX conducted the research, performed the statistical analyses, and interpreted the data; LL and CL wrote the manuscript; and CL had primary responsibility for the final content. All the authors critically reviewed the manuscript and approved the final version.

## Conflict of Interest Statement

The authors declare that the research was conducted in the absence of any commercial or financial relationships that could be construed as a potential conflict of interest.

## Footnotes

^1^http://www.uniprot.org/.

^2^http://bioinformatics.psb.ugent.be/webtools/Venn/.
